# An Unusual Cause of Intestinal Obstruction in a Young Adult Patient: Inflammatory Fibroid Polyp

**DOI:** 10.1155/2017/3675848

**Published:** 2017-07-05

**Authors:** Meryem Rais, Hafsa Chahdi, Mohammed Elfahssi, Abderrahmane Albouzidi, Mohamed Oukabli

**Affiliations:** ^1^Department of Pathology, Faculty of Medicine and Pharmacy and Mohammed V Military Hospital, Mohammed V University, Rabat, Morocco; ^2^Department of Digestive Surgery, Faculty of Medicine and Pharmacy and Mohammed V Military Hospital, Mohammed V University, Rabat, Morocco

## Abstract

Inflammatory fibroid polyps are uncommon benign lesions that originate in the submucosa of the gastrointestinal tract. The stomach and the ileum are the most commonly affected sites. Although inflammatory fibroid polyp is one of the rare conditions leading to intestinal obstruction in adults, it should be considered as a possible diagnosis in obstructive tumors of the small bowel causing intussusceptions. We present one case of inflammatory fibroid polyp as a rare cause of intussusception in a young adult patient.

## 1. Introduction

Inflammatory fibroid polyps (IFPs) are rare, benign lesions arising from the submucosa of the gastrointestinal tract. The average age of presentation is the 6th to 7th decade of life. Most cases occur in the stomach, followed by the small bowel, and, more rarely, the large bowel, duodenum, gallbladder, and oesophagus [1, 2]. The clinical symptoms vary depending on the location of the lesion. In the small bowel, IFPs rarely cause intussusceptions [3]. We report an unusual case of ileoileal intussusception caused by an IFP, whose diagnosis was confirmed by immunohistochemistry.

## 2. Case Report

**Figure 1:**
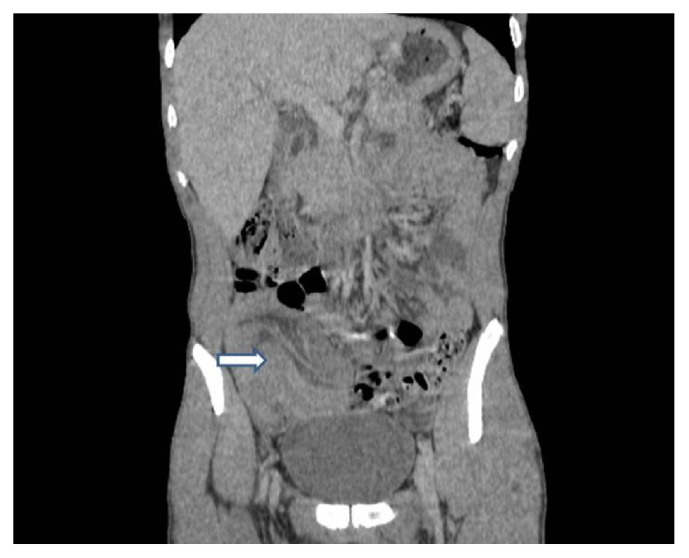
Coronal CT scan of the abdomen and pelvis showing a pseudokidney mass (arrow).

**Figure 2:**
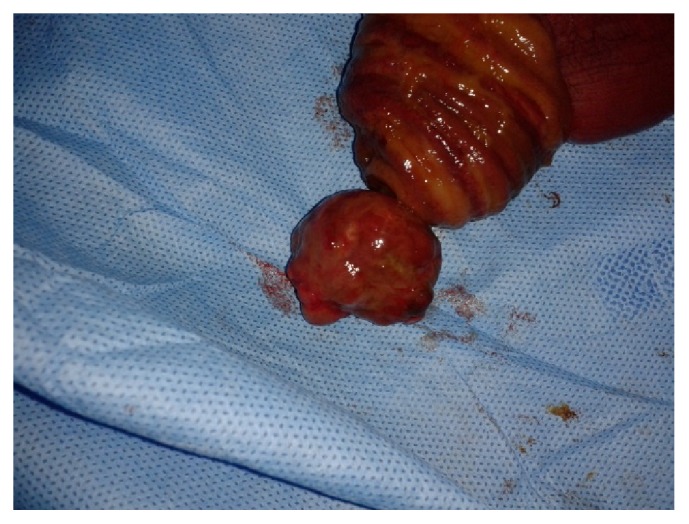
Macroscopic appearance of the resected specimen, showing a 3 cm pedunculated polyp.

**Figure 3:**
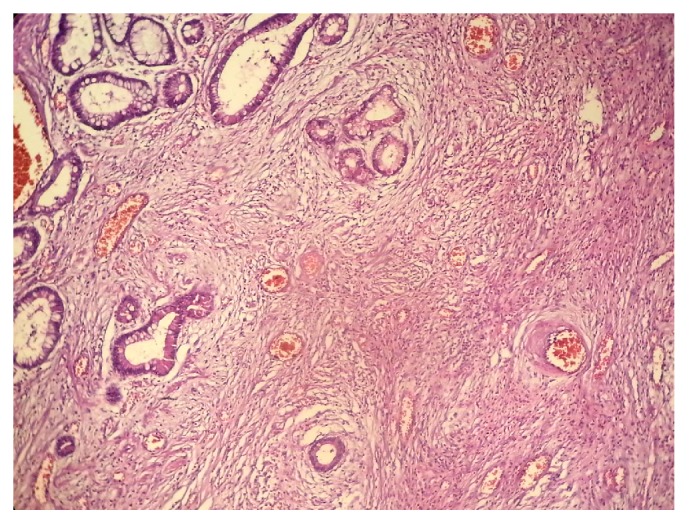
Hematoxylin and eosin (H&E) stain demonstrating a mucosal and submucosal spindle cell proliferation showing an “onion-skin” disposition around the abundant blood vessels.

**Figure 4:**
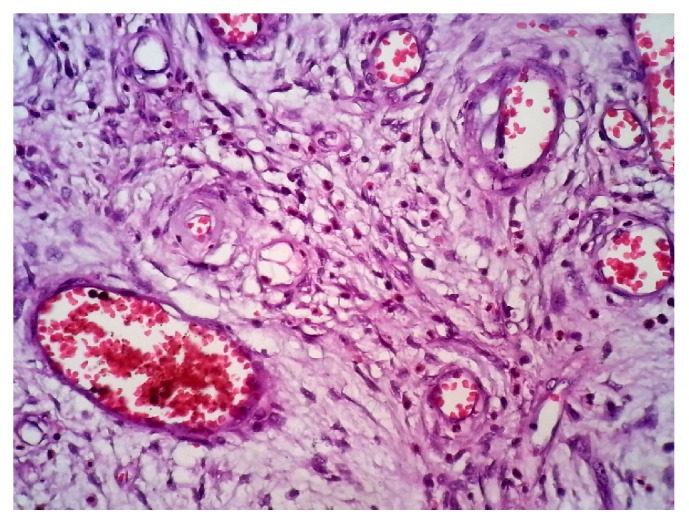
H&E stain demonstrating an associated abundant inflammatory infiltrate dominated by eosinophils.

**Figure 5:**
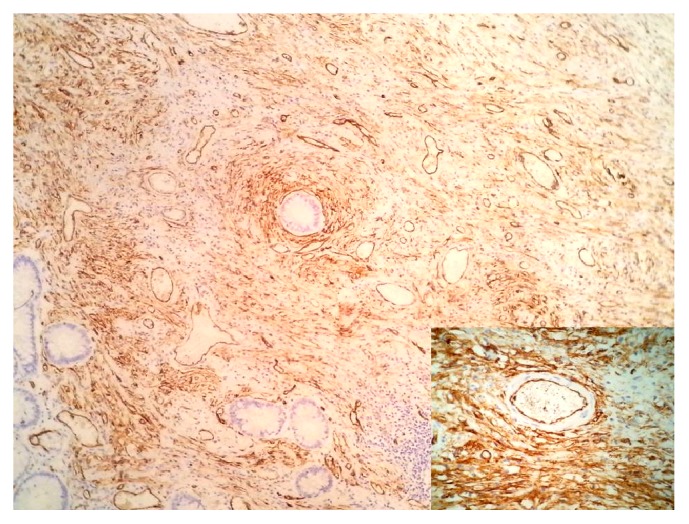
CD34 immunostaining showing diffuse positivity in the spindle cells.

A 22-year-old man presented to our hospital with acute abdominal pain, vomiting, and nausea. He had a history of intermittent constipation and weight loss in the previous year. He had no previous surgical intervention. Physical examination found abdominal distention. Abdominal X-ray showed dilated small bowel segments with marked small bowel air-fluid levels. Computerized tomography of the abdomen demonstrated a thickening of small bowel loops with a pseudokidney pattern, suggestive of intussusception (Figure 1). Exploratory laparotomy showed an ileoileal intussusception completely obstructing the ileal lumen. Segmental resection of the obstructed ileal segment and end-to-end anastomosis were performed. Macroscopic examination of the resected ileal segment found a 3 × 3 × 3 cm firm pedunculated polyp projecting into the bowel lumen (Figure 2). Microscopic examination revealed a mucosal and submucosal proliferation of loose spindle cells arranged in short fascicles or whorled structures, often in an “onion-skin” disposition around the abundant blood vessels (Figure 3). There was an associated abundant inflammatory infiltrate comprising mainly eosinophils (Figure 4). On immunohistochemical studies, the spindle cells were diffusely positive for CD34 (Figure 5) and negative for CD117. The morphological features were typical of IFP and the immunoprofile was consistent with this diagnosis.

A diagnosis of IFP of the ileum was made. The patient was discharged following an uneventful postoperative recovery.

## 3. Discussion

Inflammatory fibroid polyp (IFP) is an uncommon benign lesion of the gastrointestinal tract [4]. It was first described by Vanek in 1949 as an eosinophilic submucosal granuloma [5]. The term inflammatory fibroid polyp was later introduced by Helwig and Ranier [6], suggesting an inflammatory nature of the lesion. However, recent studies have discovered that IFPs harbor mutations of PDGFRA gene, this being in favor of a neoplastic origin of IFPs [7–9].

These tumors can be found throughout the gastrointestinal tract, but the most common site is the gastric antrum, followed by the small bowel, colorectal region, gallbladder, esophagus, duodenum, and appendix [1]. They can affect any age group, but peak incidence is between the sixth and seventh decades, and there is a slight predominance in men [1]. Presenting symptoms depend on the size of the tumor and its localization in the gastrointestinal tract. In the small bowel, IFPs can cause chronic episodes of abdominal pain, lower gastrointestinal bleeding, anemia, and, more rarely, intestinal obstruction due to intestinal intussusception [10]. These clinical characteristics are similar to those of our patient, except for the age which is 22 years.

Intussusception is an invagination of a proximal part of bowel along with its mesentery into an immediately adjacent segment [1]. This condition is uncommon in adults, accounting for only 1%–5% of all cases of intestinal obstruction [11]. Preoperative diagnosis of intussusceptions, as in this patient, is challenging. Clinically, an abdominal mass can be found. Ultrasonography is the imaging exam of choice; classical features of intussusceptions comprise a target sign in transverse view and a pseudokidney sign in longitudinal view [12]. On computed tomography, a bowel-within-bowel configuration, with fat and vessels compressed between the walls of the small bowel, is pathognomonic of intussusceptions [11]. Seventy to ninety percent of all adult intussusceptions happen as a result of a malignant or benign lesion usually appearing at the head of the invagination [13]. Benign lesions that can induce intussusception in the small bowel include lipomas, hamartomatous and inflammatory polyps, and adenomas. Malignant tumors include lymphomas, gastrointestinal stromal tumors (GISTs), and adenocarcinomas [13]. Although imaging examinations are required to identify the intussusceptions as the cause of obstruction, pathological confirmation is needed for the definitive diagnosis of IFPs. Macroscopically, they present as pedunculated or sessile polyps, measuring between 2 and 5 cm in most cases (extremes 0,2–20 cm), and they are usually submucosal and protrude into the bowel lumen [1]. Microscopically, IFPs are made of bland spindled stromal cells admixed to an inflammatory infiltrate consisting mainly of eosinophils, with numerous small blood vessels in an edematous background. The spindle cells are often arranged concentrically around blood vessels, and this is referred to as “onion skinning” [14, 15]. The lesions are usually centered on the submucosa, and they rarely extend to the muscularis propria and exceptionally reach the serosa [16]. On immunohistochemistry, these tumors show positive staining with CD34 and vimentin and variable staining with smooth muscle actin. They are negative for CD117, S100, and ALK1 [14, 17, 18]. IFPs should be differentiated from other spindle cell tumors of the gastrointestinal tract, which include GISTs, schwannomas, and inflammatory myofibroblastic tumors (IMTs) [8, 18]. This distinction is more challenging in the absence of the characteristic microscopic features of IFPs and requires immunohistochemistry. Gastrointestinal stromal tumors are positive with CD117 while IFPs are not [1]. IMTs have an inflammatory infiltrate with more lymphoid cells and less eosinophils than IFPs, and they express ALK1 while IFTs do not [18]. Schwannomas are positive for S100, which differentiates them from IFTs [8].

The optimal surgical management of intussusceptions in adult patients depends on the presence of a malignancy or manifestations of ischemia of the involved bowel. Intussusceptions in the small intestine result from malignant lesions in 1% to 47% of cases, and the majority of these lesions are metastatic. Consequently, recent reports have recommended initial reduction of externally viable small bowel prior to resection. The incidence of malignancy as the cause of ileocolic and colocolic intussusceptions ranges from 43% to 100%, most of these lesions appear as primary lesions, and a resection without reduction is therefore recommended on those cases [1].
